# Supplementary material for: Neutrophils dominate in opsonic phagocytosis of P. falciparum blood-stage merozoites and protect against febrile malaria
Source: Commun Biol. 2021 Aug 19;4:984. doi: 10.1038/s42003-021-02511-5 (PMC8376957; doi:10.1038/s42003-021-02511-5)
Supplement: Supplementary file 2 — Supplementary information [file 42003_2021_2511_MOESM2_ESM.pdf]

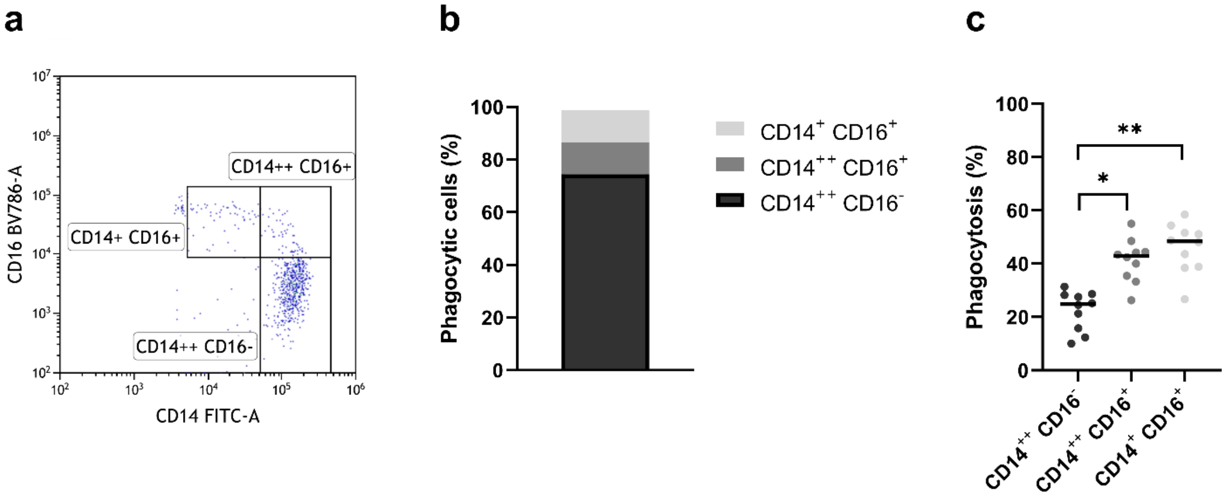

Supplementary Figure 1. Phagocytosis of merozoites by monocyte subsets.

Ethidium bromide stained merozoites were opsonized with a pool of immune plasma prior to incubation with PBLs from Danish blood donors (n=10) for 30 min at 37°C for phagocytosis to occur. **A**) Monocytes in the PBL preparations were classified as classical ($CD14^{++}CD16^{-}$), intermediate ($CD14^{++}CD16^{+}$), or non-classical ($CD14^{+}CD16^{+}$) by flow cytometry. **B**) Median percentage of monocyte subsets in the EtBr-positive monocyte fraction. **C**) Percent phagocytosis of merozoites by different monocyte subsets in all monocytes (EtBr-positive and negative). Lines represent medians. P values were determined by Friedman test and Dunn's multiple comparisons test; asterisks represent P values (*, P < 0.05; **, P < 0.01).

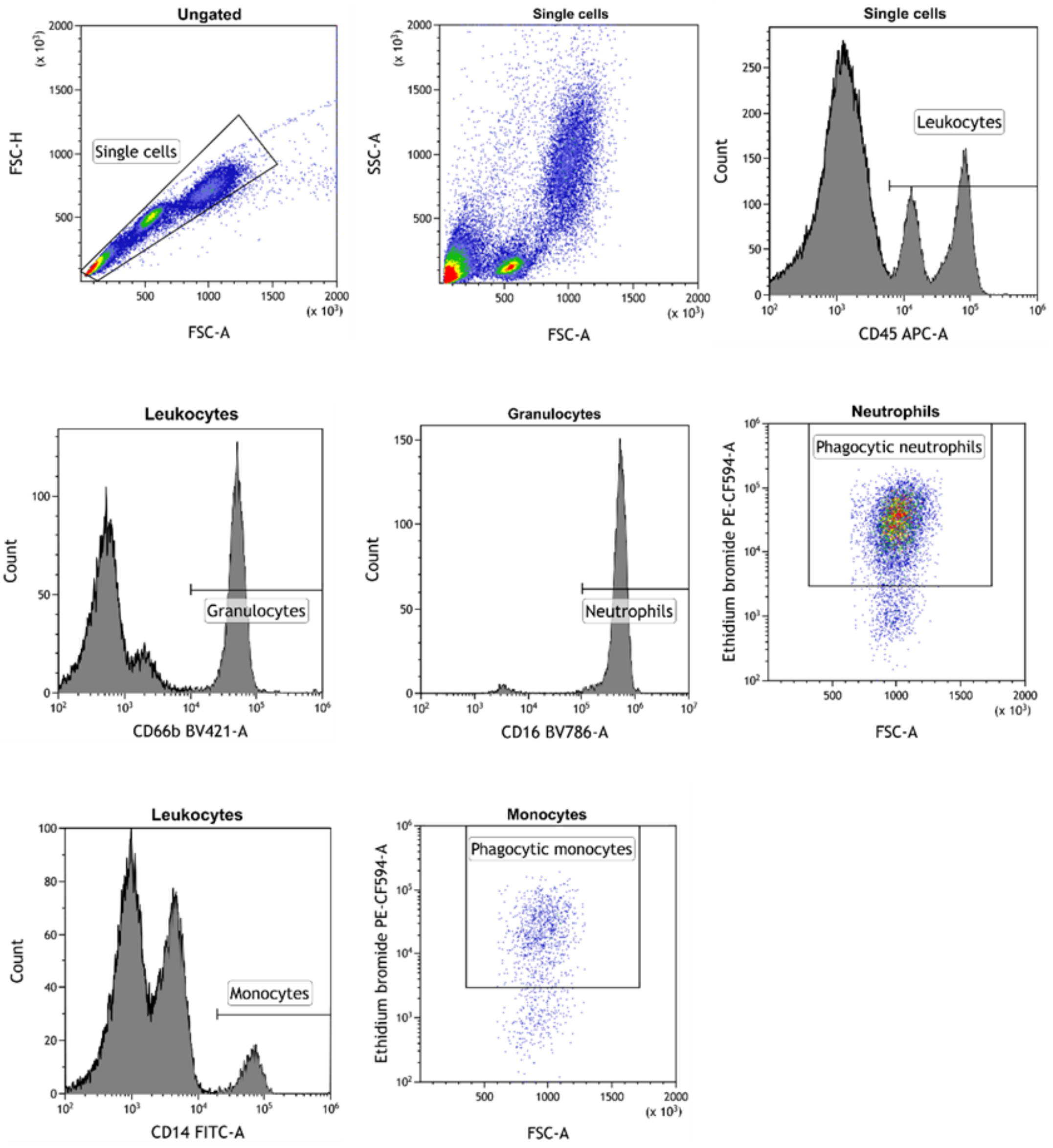

Supplementary Figure 2. Gating strategy for the evaluation of merozoite phagocytosis by neutrophils and monocytes.

Leukocytes were isolated from peripheral blood of healthy donors by centrifugation followed by hypotonic lysis of erythrocytes. Leukocytes were incubated with ethidium bromide-stained merozoites for 30 min at 37°C to allow phagocytosis to occur. The panels show the gating strategy

to assess phagocytosis by neutrophils defined as CD45+/CD66b+/CD16+ cells and monocytes defined as CD45+/CD14+ cells. Ethidium bromide signal was measured in the PE-CF594 channel (610/20 nm detector).

Supplementary Table 1 Demographics of study participants, febrile malaria status during follow-up and functional assay performed.

	**Subgroup**	**Ghana**	**India**
Total number		140	121
Age (years)		1-12	3-60
Sex	Female	67 (47.9%)	59 (48.8%)
	Male	73 (52.1%)	62 (51.2%)
Bed net use	Yes	45 (32.1%)	14 (11.6%)
	No	95 (67.9%)	107 (88.4%)
Sickle cell	Negative	118 (84.3%)	Not available
	Positive	22 (15.7%)	Not available
Follow-up febrile malaria status	Susceptible	81 (57.9%)	50 (41.3%)
	Protected	59 (42.1%)	71 (58.7%)
Phagocytosis assay performed	Peripheral blood leukocytes	140	121
	Purified neutrophils	140	Not available
