# Supplementary material for: Neutrophils dominate in opsonic phagocytosis of P. falciparum blood-stage merozoites and protect against febrile malaria
Source: Commun Biol. 2021 Aug 19;4:984. doi: 10.1038/s42003-021-02511-5 (PMC8376957; doi:10.1038/s42003-021-02511-5)
Supplement: Supplementary file 3 — Description of Additional Supplementary Files [file 42003_2021_2511_MOESM3_ESM.pdf]

Description of Additional Supplementary Files

File name: Supplementary Data 1

Description: Data underlying the graphs in the main figures.
