# Supplementary material for: Neutrophils dominate in opsonic phagocytosis of P. falciparum blood-stage merozoites and protect against febrile malaria
Source: Commun Biol. 2021 Aug 19;4:984. doi: 10.1038/s42003-021-02511-5 (PMC8376957; doi:10.1038/s42003-021-02511-5)
Supplement: Supplementary file 5 — Reporting Summary [file 42003_2021_2511_MOESM5_ESM.pdf]

nature research

Corresponding author(s): Michael Theisen

Last updated by author(s): Jun 22, 2021

Reporting Summary

Nature Research wishes to improve the reproducibility of the work that we publish. This form provides structure for consistency and transparency in reporting. For further information on Nature Research policies, see our Editorial Policies and the Editorial Policy Checklist.

Statistics

For all statistical analyses, confirm that the following items are present in the figure legend, table legend, main text, or Methods section.

n/a	Confirmed	
☐	☒	The exact sample size (*n*) for each experimental group/condition, given as a discrete number and unit of measurement
☐	☒	A statement on whether measurements were taken from distinct samples or whether the same sample was measured repeatedly
☐	☒	The statistical test(s) used AND whether they are one- or two-sided<br>*Only common tests should be described solely by name; describe more complex techniques in the Methods section.*
☐	☒	A description of all covariates tested
☒	☐	A description of any assumptions or corrections, such as tests of normality and adjustment for multiple comparisons
☐	☒	A full description of the statistical parameters including central tendency (e.g. means) or other basic estimates (e.g. regression coefficient) AND variation (e.g. standard deviation) or associated estimates of uncertainty (e.g. confidence intervals)
☐	☒	For null hypothesis testing, the test statistic (e.g. *F*, *t*, *r*) with confidence intervals, effect sizes, degrees of freedom and *P* value noted<br>*Give P values as exact values whenever suitable.*
☒	☐	For Bayesian analysis, information on the choice of priors and Markov chain Monte Carlo settings
☒	☐	For hierarchical and complex designs, identification of the appropriate level for tests and full reporting of outcomes
☐	☒	Estimates of effect sizes (e.g. Cohen's *d*, Pearson's *r*), indicating how they were calculated

Our web collection on statistics for biologists contains articles on many of the points above.

Software and code

Policy information about availability of computer code

Data collection	No software was used.
Data analysis	Prism version 9<br>R version 3.6.2 with the package geepack

For manuscripts utilizing custom algorithms or software that are central to the research but not yet described in published literature, software must be made available to editors and reviewers. We strongly encourage code deposition in a community repository (e.g. GitHub). See the Nature Research guidelines for submitting code & software for further information.

Data

Policy information about availability of data

All manuscripts must include a data availability statement. This statement should provide the following information, where applicable:

- Accession codes, unique identifiers, or web links for publicly available datasets
- A list of figures that have associated raw data
- A description of any restrictions on data availability

The data that support the findings of this study are available from the corresponding author upon reasonable request and pending agreement from relevant ethics committees for clinical data.

Field-specific reporting

Please select the one below that is the best fit for your research. If you are not sure, read the appropriate sections before making your selection.

☒ Life sciences ☐ Behavioural & social sciences ☐ Ecological, evolutionary & environmental sciences

For a reference copy of the document with all sections, see nature.com/documents/nr-reporting-summary-flat.pdf

Life sciences study design

All studies must disclose on these points even when the disclosure is negative.

Sample size	In total, the longitudinal cohort studies in Ghana and India included 798 and 945 individuals respectively. To avoid mislabeling non-exposed individuals as protected, only participants who were definitely exposed to Plasmodium falciparum were included in the analyses. Therefore, we included 140 and 121 participants from the Ghanaian and Indian cohorts, respectively, whose blood smear was positive for malaria parasites and had enough plasma volume for the assays.
Data exclusions	No data were excluded from the analyses
Replication	The experimental findings presented in Figures 1-3 and in supplementary figures were successfully reproduced. However, the findings presented in Figure 4 and 5 could not be replicated due to limited availability of plasma samples.
Randomization	Randomization and blinding not applicable for these longitudinal cohort studies.
Blinding	Randomization and blinding not applicable for these longitudinal cohort studies.

Reporting for specific materials, systems and methods

We require information from authors about some types of materials, experimental systems and methods used in many studies. Here, indicate whether each material, system or method listed is relevant to your study. If you are not sure if a list item applies to your research, read the appropriate section before selecting a response.

Materials & experimental systems

n/a	Involved in the study	
☐	☒	Antibodies
☒	☐	Eukaryotic cell lines
☒	☐	Palaeontology and archaeology
☒	☐	Animals and other organisms
☐	☒	Human research participants
☒	☐	Clinical data
☒	☐	Dual use research of concern

Methods

n/a	Involved in the study	
☒	☐	ChIP-seq
☐	☒	Flow cytometry
☒	☐	MRI-based neuroimaging

Antibodies

Antibodies used	FITC-conjugated anti-human CD14; clone TuK4; Thermo Fisher Scientific MA1-82074; Lot 149365<br>BV786-conjugated anti-human CD16; clone 3G8; BD Biosciences 563690; Lot 0346473<br>APC-conjugated anti-human CD45; clone HI30; BD Biosciences 555485; Lot 9291059<br>BV421 anti-human CD66b; clone G10F5; BD Biosciences 562940; Lot 1025923<br>anti-human CD16; clone 3G8; BD Biosciences 555404; Lot 9290257<br>anti-human CD32; clone FLI8.26; BD Biosciences 555447; Lot 9322756<br>anti-human CD64; clone 10.1; BD Biosciences 555525; Lot 0286490
Validation	Antibodies are routinely tested for their application for flow cytometry as stated in the manufacturer's website and the antibodies' technical data sheet. Each antibody has been used in previous studies and the relevant citations can be found in the manufacturer's website and the technical data sheet for each antibody.

Human research participants

Policy information about studies involving human research participants

Population characteristics	The participants from Ghanaian cohort (n=140) were distributed across six villages as follows: Asutsuare (39), Avakpo (10), Kewum (36), Mafikorpe (2), Osuwem (32) and Volivo (21). A total of 74 were ≤ 5 years of age and 22 (15.7%) were sickle cell positive. Twenty six (18.6%), 9 (6.4%), 35 (25%) and70 (50%) participants had blood group A, AB, B and O, respectively. Children who used bed net constituted 32.1%. Sixty seven of study participants were females.

	The participants from Indian cohort (n=121) were distributed across five Hamlets as follows: Dumargarhi(33), Jarwadih Munda (11), Karamtungri (14), Naya Torang (48) and Purana Torang (15). The study participants predominantly consisted of the Mnuda (n= 55, 45.5%), Oraon (n=25, 20.7%) and Kachhap (n=17, 14%) ethnic groups. The age of the participants ranged from 3- 60 years and 59 (48.8%) of them were females. Most of the study subjects resided in mud houses (n=112, 92.6%) and only a small fraction used bed nets (n=14, 11.6%). Thirty eight (31.4%) and 32 (26.4%) participants had primary and middle school education, respectively. Thirty one (25.6%) did not had any school education.
Recruitment	At enrollment villagers in both the Ghana and India were informed about the purpose of the study and an informed consent was obtained from study participants or their guardians before enrollment in the study. The chance of any kind of bias which may influences the outcome of the results had been avoided by random inclusion of voluntary participants regardless of their gender, ethnicity, village, education, etc.<br>Samples from anonymous Danish blood donors (aged 18 to 60 years) were obtained at Copenhagen University Hospital. These individuals are resident of central Copenhagen and provided written consent to have a small portion of their blood stored anonymously and used for research purposes.
Ethics oversight	The Ghanaian longitudinal cohort study was approved by the Institutional Review Board of Noguchi Memorial Institute for Medical Research of the University of Ghana, Accra, Ghana and the Indian study was approved by the Institutional Ethics Committee of the National Institute of Malaria Research, Indian Council of Medical Research, New Delhi, India.<br>Ethical approval for Danish blood donor samples was given by the Scientific Ethics Committee of Copenhagen and Frederiksberg, Denmark.

Note that full information on the approval of the study protocol must also be provided in the manuscript.

Flow Cytometry

Plots

Confirm that:

☒ The axis labels state the marker and fluorochrome used (e.g. CD4-FITC).

☒ The axis scales are clearly visible. Include numbers along axes only for bottom left plot of group (a 'group' is an analysis of identical markers).

☐ All plots are contour plots with outliers or pseudocolor plots.

☒ A numerical value for number of cells or percentage (with statistics) is provided.

Methodology

Sample preparation	Peripheral blood leukocytes were isolated from whole blood samples by centrifugation followed by red blood cell lysis. Leukocytes were transferred to 96-well U-bottom plates containing 6x10^4 cells in 100 µl of cell medium per well. Opsonized, ethidium bromide-stained merozoites were added to the leukocyte preparation and incubated for 30 min (unless stated otherwise) at 37 °C and 5 % CO2. Plates were centrifuged in a pre-chilled centrifuge and washed twice with ice-cold FACS buffer to stop phagocytosis. Cells were resuspended in 200 µl of cold FACS buffer and incubated for 1 hour at 4°C with 1:1600 FITC anti-human CD14 (clone TuK4; Thermo Fisher Scientific MA1-82074), 1:400 BV786 anti-human CD16 (clone 3G8; BD Biosciences 563690), 1:800 APC anti-human CD45 (clone HI30; BD Biosciences 555485), and 1:800 BV421 anti-human CD66b (clone G10F5; BD Biosciences 562940) antibodies. After washing thrice with FACS buffer, sample fluorescence was quantified with a CytoFLEX S (Beckman Coulter Life Sciences). Phagocytosis was determined by measuring the ethidium bromide fluorescence using the 610/20 nm detector.
Instrument	CytoFLEX S; Beckman Coulter B75442
Software	Kaluza Analysis Software version 2.1
Cell population abundance	The relative abundance of leukocytes is shown in Figure 1A. On average, samples consisted of 6% monocytes, 59% neutrophils, 32% lymphocytes, and 3% eosinophils and basophiles.<br>Highly purified neutrophils (>99%) were also used to test the association of opsonic phagocytosis with protection. Neutrophils were defined as CD45+/CD66b+/CD16+ cells.
Gating strategy	Single events were gated by plotting FSC-H and FSC-A.<br>Then, leukocytes were gated based on positive CD45 signal.<br>Granulocytes were gated as CD66b positive events in the leukocyte fraction.<br>Neutrophils were defined as the CD16 positive fraction of granulocytes.<br>Monocytes were gated based on CD14 expression.<br>Monocytes subsets were defined as classical (CD14++CD16-), intermediate (CD14++CD16+), or non-classical (CD14+CD16+).<br>Phagocytic cells were gated based on ethidium bromide signal (measured in the PE-CF594 channel).

☒ Tick this box to confirm that a figure exemplifying the gating strategy is provided in the Supplementary Information.
