# Supplementary material for: Neutrophils dominate in opsonic phagocytosis of P. falciparum blood-stage merozoites and protect against febrile malaria
Source: Commun Biol. 2021 Aug 19;4:984. doi: 10.1038/s42003-021-02511-5 (PMC8376957; doi:10.1038/s42003-021-02511-5)
Supplement: Supplementary file 6 — File checklist [file 42003_2021_2511_MOESM6_ESM.pdf]

File checklist

Should you have any questions regarding this checklist, please contact the relevant *Communications* journal.

Item	Permissible file format	File name on manuscript tracking system	File type on manuscript tracking system	Notes	
Editorial Requests Table	.doc, .docx	Editorial Requests Table	Related Manuscript File	Please provide a copy of the Editorial Requests Table supplied with our decision letter, with all changes made in response to our requests detailed in the right-hand column.	☒
Cover letter (optional)	.doc, .docx, .pdf	Cover letter	Author Cover Letter	Outline any additional changes to the manuscript.	☒
Author responses	.doc, .docx, .pdf	Response to Referees	Rebuttal Letter	Provide your point-by-point response to any issues raised by our reviewers (please include the reviewers' comments in this document).	☒
Article File	.doc, .docx, .tex	Article File	Article (NOT revised manuscript - marked up)	Main manuscript file must be in Microsoft Word or LaTeX format.<br><br>LaTex and Tex article source files must be accompanied by the compiled PDF for reference. The bibliography must be submitted separately (as a .bib file) or contained within the .tex file.	☒
Main Figure File(s)	.psd, .ai, .eps, .tiff, .jpg, .pdf, .ps, .gif, .ppt, .pptx, .png, .bmp, .vsd, .cdx, .svg or .emf	Figure 1, Figure 2, etc.	Figure	Each Figure must be provided as a separate file at a minimum resolution of 300 dpi at final size. Figures must be supplied whole, with all panels included in a single document. Figures appear at 9 or 18 cm width (1 or 2 columns respectively).<br><br>Captions must not be included in the Figure files. Figure captions must instead be included within the main manuscript file, grouped together at the end of the document.<br><br>Figures must be in file type .psd, .ai, .eps, .tiff, .jpg, .pdf, .ps, .gif, .ppt, .pptx, .png, .bmp, .vsd, .cdx, .svg or .emf. We recommend using vectographic formats as these lead to higher resolution figures.<br><br>We strongly discourage the use or adaptation of previously published images (including figures from the literature, stock photos, clip art or commercial satellite and map data), but if this is unavoidable, you must request the necessary rights documentation to re-use such material from the relevant copyright holders and submit this to us alongside your manuscript. An appropriate permissions statement must be present in the relative figure caption for any third-party images.<br><br>If individuals are identifiable in images, their written permission must be provided.	☒
Main Table(s)	.doc, .docx, .tex	Article File	Article	Included within the main Article File in word-editable format.<br><br>Tables should be grouped together at the end of the main manuscript file.	☐
Boxes (Reviews/ Perspectives only)	.doc, .docx, .tex	Box 1, Box 2, etc.	Article	Included in the main Article File in word-editable format, or uploaded as a separate Word or TeX file under the file type 'Article'.	☐
Supplementary Information	.pdf	Supplementary Information	Supplemental Material	Any Supplementary Figures, Tables, Methods, Notes, Discussion and References must be provided in a single separate file in PDF format.<br><br>We recommend limiting the size of your Supplementary Information file to 50MB.<br><br>** Please note that Supplementary Information cannot be changed after the paper has been accepted **	☒
Supplementary Data	.csv, .xlsx, .txt, .zip, .cif	Supplementary Data 1, Supplementary Data 2, etc.	Data Sets	Any Supplementary Data files should be supplied separately and should be labelled as Supplementary Data 1, etc. Legends for these should be given in the Editorial Requests Table (and not in the main Supplementary Information file).<br><br>We recommend limiting the size of each Supplementary file to 50MB.<br><br>** Please note that Supplementary Information cannot be changed after the paper has been accepted **	☒

Item	Permissible file format	File name on manuscript tracking system	File type on manuscript tracking system	Notes	
Supplementary Audio	.avi, .mp2, .wav, .mp3	Supplementary Audio 1, Supplementary Audio 2, etc.	Supplemental Material	Any Supplementary Audio files should be supplied separately and should be labelled as Supplementary Audio 1, etc. Legends for these should be given in the Editorial Requests Table (and not in the main Supplementary Information file).<br><br>We recommend limiting the size of each Supplementary file to 50MB.<br><br>** Please note that Supplementary Information cannot be changed after the paper has been accepted **	☐
Supplementary Movies	.mp4, .mpeg, .flv, .3gp, .m4v, .mts, .mxf, .mpg, .mov, .m2p, .gif, .wmv,	Supplementary Movie 1, Supplementary Movie 2, etc.	Video	Any Supplementary Movie files should be supplied separately and should be labelled as Supplementary Movie 1, etc. Legends for these should be given in the Editorial Requests Table (and not in the main Supplementary Information file).<br><br>We recommend limiting the size of each Supplementary file to 50MB.<br><br>** Please note that Supplementary Information cannot be changed after the paper has been accepted **	☐
Supplementary Software	.zip	Supplementary Software 1, Supplementary Software 2, etc.	Supplemental Material	Any Supplementary Software files should be supplied separately and should be labelled as Supplementary Software 1, etc. Legends for these should be given in the Editorial Requests Table (and not in the main Supplementary Information file).<br><br>Supplementary Software must be supplied as a ZIP file.<br><br>We recommend limiting the size of each Supplementary file to 50MB.<br><br>** Please note that Supplementary Information cannot be changed after the paper has been accepted **	☐
Life sciences reporting summary	.pdf	Reporting Summary	Supplemental Material	For life science manuscripts, a final version of the life sciences reporting summary.<br>https://www.nature.com/documents/nr-reporting-summary.pdf<br><br>The reporting summary will be published alongside your manuscript and therefore it needs to accurately represent your work. Please take a close look at the reporting summary and make sure that everything is completed correctly. In the section "Reporting for specific materials, systems and methods", you need to tick a box for each item, according to whether or not it applies, which will result in irrelevant sections becoming hidden. In the subsequent sections no box should be left blank or completed as N/A, including when the response is negative. Also, please make sure to include your name and date at the top of the document.	☒
Solar cells reporting summary	.pdf	Solar cells reporting summary	Supplemental Material	For solar cell manuscripts, a final version of the solar cells reporting summary.<br>https://www.nature.com/documents/nr-photovoltaic-reporting.pdf	☐
Lasing reporting summary	.pdf	Lasing reporting summary	Supplemental Material	For lasing manuscripts, a final version of the lasing reporting summary.<br>https://www.nature.com/documents/nr-lasing-reporting.pdf	☐
Editorial policy checklist	.pdf	Editorial policy checklist	Related Manuscript File	For all primary research articles, a final version of the editorial policy checklist.<br>https://www.nature.com/documents/nr-editorial-policy-checklist.zip<br>Please note that this form is a dynamic 'smart pdf' and must therefore be downloaded and completed in Adobe Reader. Clicking this link will download a zip file containing the pdf.	☒
Suggested feature image	.jpg, .pdf, .gif, .tiff, .psd	Featured image	Related Manuscript File	If you wish, an interesting image (but not an illustration or schematic) for consideration as a 'Featured Image' on the journal homepage. The file should be 1200x675 pixels in RGB format and should be uploaded as 'Related Manuscript File'. In addition to our home page, we may also use this image (with credit) in other journal-specific promotional material. If you submit a suggested featured image, please also include a completed image License to Publish form (also upload as 'Related Manuscript File', with file name 'Featured image LTP').	☐
